# Studying weak inter­actions in crystals at high pressures: when hardware matters

**DOI:** 10.1107/S205698901800470X

**Published:** 2018-04-17

**Authors:** Boris A. Zakharov, Zoltan Gal, Dyanne Cruickshank, Elena V. Boldyreva

**Affiliations:** a Institute of Solid State Chemistry and Mechanochemistry, Siberian Branch of the Russian Academy of Sciences, Kutateladze Str. 18, Novosibirsk, 630128, Russian Federation; b Novosibirsk State University, Pirogova Str. 2, Novosibirsk, 630090, Russian Federation; c Rigaku Oxford Diffraction, Monument Park, Chalgrove, OX44 7RW, England

**Keywords:** crystal structure, high pressure, weak inter­actions

## Abstract

The quality of structural models for 1,2,4,5-tetra­bromo­benzene (TBB), C_6_H_2_Br_4_, based on data collected from a single crystal in a diamond anvil cell at 0.4 GPa *in situ* using two different diffractometers belonging to different generations have been compared, together with the effects of applying different data-processing strategies.

## Abstract 2

The quality of structural models for 1,2,4,5-tetra­bromo­benzene (TBB), based on data collected from a single crystal in a diamond anvil cell at 0.4 GPa *in situ* using two different diffractometers belonging to different generations have been compared, alongside the effects of applying different data-processing strategies.

## Introduction   

High-pressure data are widely used for the study of inter­molecular inter­actions in crystals. In particular, high pressure can probe inter­actions and their role in stabilizing structures and their evolution across a variety of structural transformations: anisotropic structural distortion, polymorphic transitions and chemical reactions (Katrusiak, 1991 ▸; Boldyreva, 2008 ▸; Resnati *et al.*, 2015 ▸; Yan *et al.*, 2018 ▸; Parois *et al.*, 2010 ▸). The quality of diffraction data [particularly completeness and the *F^2^*/σ(*F^2^*) ratio] is critically important for obtaining reliable information on mol­ecular conformations, inter­molecular distances and even electron charge-density distribution (Veciana *et al.*, 2018 ▸; Casati *et al.*, 2017 ▸, 2016 ▸). Really impressive progress has been achieved over the last decade in obtaining more precise structural data from mol­ecular crystal structures of increasing complexity. The improvements are related, first of all, to a new design of diamond anvil cells (DACs) with larger opening angles (Sowa & Ahsbahs, 2006 ▸; Ahsbahs, 2004 ▸; Boehler, 2006 ▸; Moggach *et al.*, 2008 ▸). The improvements also include the use of 2D detectors instead of point detectors (Ahsbahs, 2004 ▸; Dubrovinsky *et al.*, 2010 ▸; Kantor *et al.*, 2012 ▸; Dawson *et al.*, 2004 ▸), as well as applying new software for sample centering, absorption correction, recognizing and excluding unwanted reflections that do not belong to the sample, data reduction, and finding the orientation matrices for several crystallites in the same diamond anvil cell (Boldyreva *et al.*, 2016 ▸; Katrusiak, 2008 ▸, 2004 ▸; Dera *et al.*, 2013 ▸; Casati *et al.*, 2007 ▸; Angel & Gonzalez-Platas, 2013 ▸). Special methods of data processing, in addition to precise experiments, now even make it possible to obtain data for charge-density studies (Veciana *et al.*, 2018 ▸; Casati *et al.*, 2017 ▸, 2016 ▸), and to follow related changes with pressure. This has been demonstrated for example by following the reduction in aromaticity of *syn*-1,6:8,13-bis­carbon­yl[14]annulene on compression (Casati *et al.*, 2016 ▸). Advances in the quality of high-pressure data for mol­ecular crystals have often been related to the use of synchrotron radiation. However, with limited access to synchrotrons, in-house experiments remain the most common type of high-pressure experiments for organic solids.

**Figure 1:**
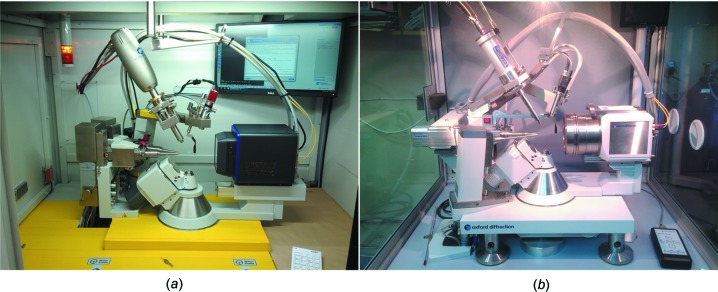
Cabinet view of diffactometers used: (*a*) XtaLAB Synergy-S Dualflex; (*b*) Oxford Diffraction Gemini R Ultra.

A new generation of laboratory diffractometers has been developed recently that makes it possible to collect data at high pressures from even small and weakly diffracting crystals. In this contribution, we present the results of a comparison of the data collected using two different diffractometers that were manufactured by the same company within a 10 year inter­val (Fig. 1 ▸). The first is an XtaLAB Synergy-S Dualflex diffractometer with Ag Kα radiation (PhotonJet-S source) and Pilatus3 X CdTe 300K hybrid photon-counting (HPC) detector from Dectris that was manufactured by Rigaku Oxford Diffraction in 2017, while the second is an Oxford Diffraction Gemini R Ultra diffractometer with Mo Kα radiation (Enhance X-ray source) and Ruby charge-coupled device (CCD) detector, manufactured by Oxford Diffraction in 2007. The main parameters characterizing the two instruments are compared in Table 1 ▸. We have collected data on the two different instruments from the same sample at the same pressure in the same DAC. We have also compared the results of applying different strategies for the data reduction.

As a sample we selected single crystals of 1,2,4,5-tetra­bromo­benzene (TBB). TBB is a well-known thermosalient compound, which exhibits large, spontaneous mechanical response across the phase transition on heating (Sahoo *et al.*, 2013 ▸; Zakharov *et al.*, 2018 ▸ and references therein). It has been shown recently that data on the high-pressure behaviour of such materials can be helpful in order to understand the origin of the thermosalient effect (Zakharov *et al.*, 2017 ▸). TBB crystallizes in the monoclinic space group *P*2_1_/*n*. Being a thermosalient material, it shows a significant mechanical response, even though the phase transition on heating is accompanied by only minute rearrangements at the mol­ecular level and only minimal changes in the inter­molecular contacts (Sahoo *et al.*, 2013 ▸; Zakharov *et al.*, 2018 ▸). This makes it important to have high-quality structural data at multiple pressure and temperature (*PT*) conditions when studying the role of the inter­molecular inter­actions in the thermosalient effect. High noise level, low data completeness, low *F^2^*/σ(*F^2^*) and data-to-number of parameters ratios can lead to the loss of most of the information related to the electron-density distribution in the crystal. When using ‘older-generation’ in-house diffractometers, low data quality can make it impossible to refine the crystal structure in even an isotropic approximation. Therefore, fine details in the orientation of anisotropic displacement parameters (ADPs) and precise values for the inter­atomic distance changes, which are of great importance for studying the mechanical response of the crystal to variations in *PT* conditions, will not be accessible. The new-generation instruments are expected to improve the quality of the diffraction data and the structural models based on the refinement of these data. At the same time, using a newer instrument alone does not guarantee a high-quality structural model. The data-processing strategy is critically important for data collected from a sample in a DAC at high pressure (Boldyreva *et al.*, 2016 ▸; Katrusiak, 2008 ▸, 2004 ▸; Dera *et al.*, 2013 ▸; Casati *et al.*, 2007 ▸; Angel & Gonzalez-Platas, 2013 ▸). These data are inevitably ‘contaminated’ by absorption of X-rays by the materials of the DAC (diamond, metal) and reflections originating from diffraction of the diamonds, gasket or the ruby calibrant. The presence of these reflections also corrupts the measured intensities of the sample reflections, either by direct overlap or because they may have an influence on the estimated background level. The aim of this study was to compare the data quality collected from the same sample in a DAC at high pressure *in situ* using diffractometers belonging to different generations. For data collected using both of the two instruments, we have used several different strategies for the data processing. The aim of this was to test the relative importance of applying different techniques for correction of the raw data for increasing the reliability and improving the quality of the structural model.

## Experimental   

Single crystals of 1,2,4,5-tetra­bromo­benzene (TBB) were prepared by slow evaporation of chloro­form solutions, using 200 mg of TBB (Sigma–Aldrich, 97%) dissolved in 9 ml of chloro­form at room temperature.
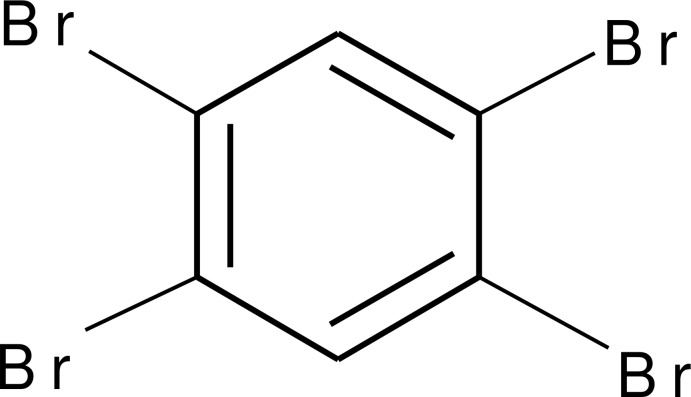


The sample was mounted in an Almax Boehler DAC (Boehler, 2006 ▸). A stainless steel sheet with an initial thickness of 200 µm was pre-indented to 100 µm and used as a gasket. The ruby fluorescence method was used for pressure calibration (Forman *et al.*, 1972 ▸; Piermarini *et al.*, 1975 ▸). A methanol–ethanol mixture (4:1) was used as hydro­static pressure-transmitting medium (Piermarini *et al.*, 1973 ▸; Angel *et al.*, 2007 ▸).

Single-crystal X-ray diffraction data were collected on the same crystal in the same DAC at a hydro­static pressure of 0.4 GPa. Data were collected using two different instruments: (1) an XtaLAB Synergy-S Dualflex diffractometer with Ag Kα radiation (PhotonJet-S source) and Pilatus3 X CdTe 300K HPC detector from Dectris (manufactured by Rigaku Oxford Diffraction in 2017), and (2) an Oxford Diffraction Gemini R Ultra diffractometer with Mo Kα radiation (Enhance X-ray source) and Ruby CCD detector (manufactured by Oxford Diffraction in 2007). Data collection, cell refinement and data reduction were performed using *CrysAlis PRO* software (Rigaku OD, 2016 ▸). Multiple strategies were tried on each instrument. Some of the strategies deliberately neglected good-practice techniques of introducing certain high-pressure data corrections in order to evaluate the extent to which this neglect can worsen the data quality.

For data collection (1), X-ray diffraction data were treated and attempts were made to refine the structure in three different ways:

(*a*) Gaussian absorption correction using *ABSORB-7* (Angel & Gonzalez-Platas, 2013 ▸) implemented in *CrysAlis PRO* software (Rigaku OD, 2016 ▸). Both crystal and DAC absorption were taken into account. The most disagreeable reflections from the sample that overlapped with diamond and gasket reflections were not excluded from the *HKL* file. All non-H atoms were refined anisotropically.

(*b*) Gaussian absorption correction using *ABSORB-7* (Angel & Gonzalez-Platas, 2013 ▸) implemented in *CrysAlis PRO* software (Rigaku OD, 2016 ▸). Both crystal and DAC absorption were taken into account. The most disagreeable reflections from the sample that overlapped with diamond and gasket reflections were excluded manually from the *HKL* file. All non-H atoms were refined anisotropically.

(*c*) Spherical absorption correction as implemented in *CrysAlis PRO* software (Rigaku OD, 2016 ▸). Only crystal absorption was taken into account. The most disagreeable reflections from the sample that overlapped with diamond and gasket reflections were manually excluded from the *HKL* file. All non-H atoms were refined anisotropically.

For data collection (2), X-ray diffraction data were treated and attempts were made to refine in six different ways:

(*d*) the same as for (*a*).

(*e*) the same as for (*b*).

(*f*) the same as for (*c*).

(*g*) the same as for (*a*), but carbon atoms were refined isotropically.

(*h*) the same as for (*b*), but carbon atoms were refined isotropically.

(*i*) the same as for (*c*), but carbon atoms were refined isotropically.

For all the refinements at high pressure, the initial crystal structure model was taken from single-crystal diffraction data at ambient conditions (Zakharov *et al.*, 2018 ▸). Refinements were carried out with *SHELXL2018/1* (Sheldrick, 2015 ▸) using *Shelxle* (Hübschle *et al.*, 2011 ▸) as the GUI without any restraints. Hydrogen-atom parameters were constrained using AFIX 43 with *U*_iso_(H) = 1.2*U*_eq_(C). *Mercury* (Macrae *et al.*, 2008 ▸), *checkCIF* */PLATON* (Spek, 2009 ▸) and *publCIF* (Westrip, 2010 ▸) were used for structure visualization, analysis and preparation of the CIF files for publication.

## Results and discussion   

Crystal data, data collection and refinement parameters are summarized in Table 2 ▸. In comparison with the older Gemini R Ultra device, used for data collection (2), the Synergy-S diffractometer, used for data collection (1), was superior for data collection. Compared to instrument (2), collection of single-crystal X-ray data on (1) was much faster (6 *vs* 32 h), with a higher *F^2^*/σ(*F^2^*) ratio (18 *vs.* 10) and data completeness (68 *vs* 58%). A higher *HKL* range allowed us to increase the number of reflections used for cell-parameter refinement by a factor of 1.5. The resulting values of the lattice parameters appear to be almost the same in the two cases: the largest difference, 0.2%, was observed for lattice parameter *b*. Standard uncertainties for the cell parameters were slightly higher for (1) than for (2). This is presumably related to the smaller 2θ values for stronger reflections owing to the use of the harder Ag *K*α radiation.

Shorter wavelengths are generally prefered for samples mounted in a DAC with a fixed window-opening size. From a data completeness point of view, this provides the same number of reflections in a narrower 2θ range. Ag *K*α radiation is therefore becoming popular for high-pressure X-ray diffraction studies (Saouane *et al.*, 2013 ▸; Saouane & Fabbiani, 2015 ▸; Granero-García *et al.*, 2017 ▸). The number of independent reflections for data collection (1) was 1.6 times greater than for (2) (893 *vs* 550), as a result of using a shorter wavelength. The more efficient HPC detector and the brighter X-ray source allowed us to measure reflection intensities with higher precision. This gave us a twofold lower *R*_int_ value for data collection (1): 0.048 for data set (*b*) *vs* 0.105 for data sets (*e*) and (*h*).

**Figure 2:**
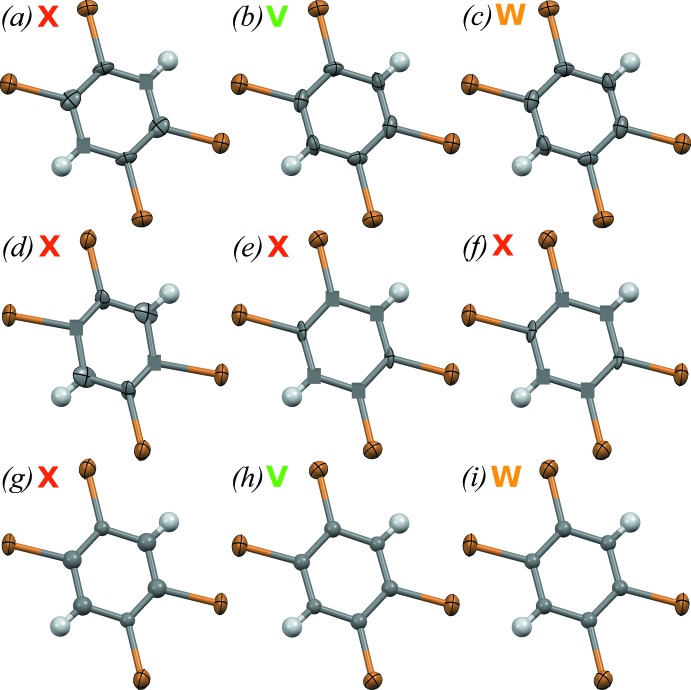
Displacement ellipsoid plots for 1,2,4,5-tetra­bromo­benzene mol­ecules obtained with different data-treatment procedures. Carbon atoms for structure refinements (*g*), (*h*) and (*i*) were refined using an isotropic approximation. Cubes show atoms with negative thermal parameters. Refinements marked **V** are preferable for publication; those marked **W** are publishable but not always preferable, and those marked **X** are not acceptable for publication (incorrect).

Displacement ellipsoid plots for the different methods of data treatment and refinement are shown in Fig. 2 ▸. Taking into account the refinement data presented in Table 2 ▸, one can conclude that the best results are provided by refinements (*b*) and (*c*), where the use of a modern device permitted a more precise and faster measurement of the intensities of the diffraction reflections. The quality of the diffraction data enabled a crystal-structure refinement in the anisotropic approximation for all non-H atoms, providing reasonable values and shapes of the displacement ellipsoids. For the refinement variant (*a*), for which the sample reflections that overlapped with diamond and gasket reflections were not excluded from the *HKL* file, the refinement did not converge, and when an anisotropic refinement was attempted a non-positive-definite atomic displacement ellipsoid was obtained for one of the carbon atoms.

For data collection (2), the refinement results were of much lower quality than those for data collection (1). As expected, the worst results were provided by refinements (*d*) and (*g*) for which the sample reflections that overlapped with the diamond and gasket reflections were not excluded from the *HKL* file. The refinement did not converge, and two of the carbon atoms were characterized by non-positive-definite ellipsoids when attempting to use an anisotropic model. Removal of the corrupted reflections from the *HKL* file did not improve refinement results. The anisotropic thermal parameters were still not adequate for the (*e*) and (*f*) refinements. Publishable refinement results in this case of impossible anisotropic refinement could be obtained in two ways: *viz*. by applying *SHELX* restraints for the thermal parameters of carbon atoms, *e.g.* SIMU and DELU, with low standard uncertainty values, or by refining the carbon atoms in an isotropic approximation, as was done for the (*h*) and (*i*) refinements.

Different absorption correction types were tested for both data collection strategies. The refinement results provided by the Gaussian and spherical absorption corrections are defined as (*b*) and (*c*), (*e*) and (*f*), (*h*) and (*i*), respectively. One can see that the *R*-factors are comparable and acceptable for both absorption-correction strategies. A potential explanation for the similarity of the Gaussian and spherical absorption correction results for data collection (1) rests in the fact that TBB is a medium-absorbing sample (*μ* is 10.33 mm^−1^ for Ag *K*α). In the case of data collection (2), TBB is much more absorbing (*μ* is 19.29 mm^−1^ for Mo *K*α radiation) but the overall data quality is low (intensities are not measured precisely) and even the good-practice procedure of applying an absorption correction does not improve data quality. Generally, it is preferable to use a Gaussian absorption correction (both for the crystal and for the DAC), especially for strongly absorbing samples since it calculates the ‘true’ transmission factors using the actual crystal and DAC geometries. For example, data sets (*b*) and (*h*), and (*e*) in the case of reasonable anisotropic thermal displacement parameters, would be the most preferable for the experimental set-up described.

## Conclusions   

In order to obtain reliable information on inter­molecular inter­actions in a crystal structure, one needs high-quality data. This is especially critical for data collected in a DAC at high pressure, when data completeness and the availability of reciprocal space are limited. A comparison of the results obtained using different instruments and different data-processing methods has illustrated that the data processing itself plays a crucial role in obtaining reliable results. At the same time, a modern instrument belonging to the new generation makes it possible to speed up data collection, increase the signal-to-noise intensity ratio and the number of observed reflections, and with shorter wavelength data completeness for a sample mounted in a DAC. Data collection for the 1,2,4,5-tetra­bromo­benzene crystal mounted in a DAC using a modern XtaLAB Synergy-S Dualflex diffractometer with Ag *K*α radiation and a Pilatus3 X CdTe 300K HPC detector took six hours, and allowed us to obtain high-quality data for an anisotropic crystal-structure refinement without any restraints.

Using the older diffractometer from the previous generation, an Oxford Diffraction Gemini R Ultra with Mo *K*α radiation and a Ruby CCD detector, did not allow us to obtain diffraction data of the same quality, even when using a higher exposure time, for which data collection took 32 h; the anisotropic refinement was possible only for the heavier bromine atoms. The carbon atoms could be refined reasonably only in an isotropic approximation, or by restraining their thermal parameters. Data completeness, *HKL* ranges and the *F^2^*/σ(*F^2^*) ratio were lower, and the *R*-factors were higher compared to the values obtained when using the modern XtaLAB Synergy-S Dualflex diffractometer described above.

Crystal-structure refinement using the same primary data set, but different data-reduction strategies has revealed that eliminating the sample reflections with wrong intensities (affected by the presence of diamond, as well as powder-diffraction rings originating from the metal gasket) is the most important correction of primary data. The exact procedure for the absorption correction was less critical in the particular case considered in this work. However, generally and especially for strong absorbers, a Gaussian absorption correction both for the crystal and the DAC data can help to increase the quality of the refinement significantly, since it calculates the ‘true’ transmission factors using the actual crystal and DAC geometries.

## Supplementary Material

Crystal structure: contains datablock(s) Ag-Absorb7-raw_a, Ag-Absorb7_b, Ag-CAsphere_c, Mo-Absorb7-raw_d, Mo-Absorb7_e, Mo-CAsphere_f, Mo-Absorb7-raw-Ciso_g, Mo-Absorb7-Ciso_h, Mo-CAsphere-Ciso_i. DOI: 10.1107/S205698901800470X/su5433sup1.cif

Supporting information file. DOI: 10.1107/S205698901800470X/su5433Ag-Absorb7-raw_asup11.cml

Structure factors: contains datablock(s) Ag-Absorb7-raw_a. DOI: 10.1107/S205698901800470X/su5433Ag-Absorb7-raw_asup2.hkl

Structure factors: contains datablock(s) Ag-Absorb7_b. DOI: 10.1107/S205698901800470X/su5433Ag-Absorb7_bsup3.hkl

Structure factors: contains datablock(s) Ag-CAsphere_c. DOI: 10.1107/S205698901800470X/su5433Ag-CAsphere_csup4.hkl

Structure factors: contains datablock(s) Mo-Absorb7-raw_d. DOI: 10.1107/S205698901800470X/su5433Mo-Absorb7-raw_dsup5.hkl

Structure factors: contains datablock(s) Mo-Absorb7_e. DOI: 10.1107/S205698901800470X/su5433Mo-Absorb7_esup6.hkl

Structure factors: contains datablock(s) Mo-CAsphere_f. DOI: 10.1107/S205698901800470X/su5433Mo-CAsphere_fsup7.hkl

Structure factors: contains datablock(s) Mo-Absorb7-raw-Ciso_g. DOI: 10.1107/S205698901800470X/su5433Mo-Absorb7-raw-Ciso_gsup8.hkl

Structure factors: contains datablock(s) Mo-Absorb7-Ciso_h. DOI: 10.1107/S205698901800470X/su5433Mo-Absorb7-Ciso_hsup9.hkl

Structure factors: contains datablock(s) Mo-CAsphere-Ciso_i. DOI: 10.1107/S205698901800470X/su5433Mo-CAsphere-Ciso_isup10.hkl

CCDC references: 1831715, 1831721, 1831714, 1831716, 1831717, 1831718, 1831719, 1831720, 1831722

Additional supporting information:  crystallographic information; 3D view; checkCIF report

## Figures and Tables

**Table 1:** Comparison of technical characteristics of the diffractometers

	XtaLAB Synergy-S Dualflex	Oxford Diffraction Gemini *R* Ultra
Radiation type	Ag *K*α	Mo *K*α
X-ray source type	PhotonJet-S source	Enhance X-ray source
Beam characteristics	0.12 mm beam	0.5 mm beam
X-ray optics	double-bounce multilayer optics	graphite monochromator
Detector model	Pilatus3 *X* CdTe 300K	Ruby
Detector type	HPC – photon counting	CCD – integrative detector
Quantum efficiency	>90%	>80%
Read-out frequency (Hz)	20	<0.3
Goniometer	four-circle Kappa goniometer (new generation)	four-circle Kappa goniometer
Data collection mode	shutterless data collection	shuttered data collection

**Table:** For all structures: C_6_H_2_Br_4_, *M*_r_ = 393.72, monoclinic, *P*2_1_/*n*, *Z* = 2. Experiments were carried out at 293 K. Crystal size 0.18 × 0.07 × 0.01 (mm). H-atom parameters were constrained. Refinements not acceptable for publication (incorrect) are highlighted in red, preferable in green, and those publishable but not always preferable are not highlighted.

	(*a*) Ag, *ABSORB-7*, raw	(*b*) Ag, *ABSORB-7*	(*c*) Ag, CA sphere
Crystal data			
*a*, *b*, *c* (Å)	3.9390 (9), 10.781 (4), 9.944 (4)	3.9390 (9), 10.781 (4), 9.944 (4)	3.9390 (9), 10.781 (4), 9.944 (4)
β (°)	100.49 (3)	100.49 (3)	100.49 (3)
*V* (Å^3^)	415.2 (2)	415.2 (2)	415.2 (2)
Radiation type	Ag *K*α, λ = 0.56087 Å	Ag *K*α, λ = 0.56087 Å	Ag *K*α, λ = 0.56087 Å
No. of reflections for cell measurement	748	748	748
θ range (°) for cell measurement	2.2–22.9	2.2–22.9	2.2–22.9
μ (mm^−1^)	10.33	10.33	10.33

Data collection [total experiment time = 6 hours, exposure time = 45 seconds, *F*_2_/σ(*F*_2_) = 18, data completeness = 68% (inf = 0.8 Å)]			
Absorption correction	Gaussian	Gaussian	Sphere
*T*_min_, *T*_max_	0.486, 0.562	0.486, 0.562	0.638, 0.645
No. of measured, independent and observed [*I* > 2σ(*I*)] reflections	2503, 893, 513	2445, 870, 496	2453, 870, 494
*R*_int_	0.048	0.048	0.050
(sin θ/λ)_max_ (Å^−1^)	0.801	0.801	0.801
Range of *h*, *k*, *l*	*h* = −5→6, *k* = −14→14, *l* = −11→12	*h* = −5→6, *k* = −14→14, *l* = −11→12	*h* = −5→6, *k* = −14→14, *l* = −11→12

Refinement			
*R*[*F* ^2^ > 2σ(*F* ^2^)], *wR*(*F* ^2^), *S*	0.047, 0.206, 1.02	0.037, 0.073, 0.93	0.037, 0.071, 0.91
No. of reflections	893	870	870
No. of parameters	46	46	46
(Δ/σ)_max_	0.014	0.001	< 0.001
Δρ_max_, Δρ_min_ (e Å^−3^)	1.55, −1.48	0.54, −0.54	0.53, −0.49

**Table:** 

	(*d*) Mo, *ABSORB-7*, raw	(*e*) Mo, *ABSORB-7*	(*f*) Mo, CA sphere
Crystal data			
*a*, *b*, *c* (Å)	3.9431 (5), 10.7566 (18), 9.964 (2)	3.9431 (5), 10.7566 (18), 9.964 (2)	3.9431 (5), 10.7566 (18), 9.964 (2)
β (°)	100.557 (15)	100.557 (15)	100.557 (15)
*V* (Å^3^)	415.47 (13)	415.47 (13)	415.47 (13)
Radiation type	Mo *K*α	Mo *K*α	Mo *K*α
No. of reflections for cell measurement	514	514	514
θ range (°) for cell measurement	2.8–22.4	2.8–22.4	2.8–22.4
μ (mm^−1^)	19.29	19.29	19.29

Data collection [total experiment time = 32 hours, exposure time = 60 seconds, *F*_2_/σ(*F*_2_) = 10, data completeness = 58% (inf = 0.8 Å)]			
Absorption correction	Gaussian	Gaussian	Sphere
*T*_min_, *T*_max_	0.361, 0.434	0.361, 0.434	0.638, 0.645
No. of measured, independent and observed [*I* > 2σ(*I*)] reflections	2177, 550, 323	2116, 531, 313	2125, 531, 319
*R*_int_	0.105	0.103	0.102
(sin θ/λ)_max_ (Å^−1^)	0.663	0.663	0.663
Range of *h*, *k*, *l*	*h* = −5→5, *k* = −12→11, *l* = −10→10	*h* = −5→5, *k* = −12→11, *l* = −10→10	*h* = −5→5, *k* = −12→11, *l* = −10→10

Refinement			
*R*[*F* ^2^ > 2σ(*F* ^2^)], *wR*(*F* ^2^), *S*	0.101, 0.347, 1.19	0.071, 0.169, 1.05	0.069, 0.157, 1.05
No. of reflections	550	531	531
No. of parameters	46	46	46
(Δ/σ)_max_	0.089	0.592	0.523
Δρ_max_, Δρ_min_ (e Å^−3^)	2.65, −2.89	1.04, −0.89	0.93, −0.83

**Table:** 

	(*g*) Mo, *ABSORB-7*, raw, C iso	(*h*) Mo, *ABSORB-7*, C iso	(*i*) Mo, CA sphere, C iso
Crystal data			
*a*, *b*, *c* (Å)	3.9431 (5), 10.7566 (18), 9.964 (2)	3.9431 (5), 10.7566 (18), 9.964 (2)	3.9431 (5), 10.7566 (18), 9.964 (2)
β (°)	100.557 (15)	100.557 (15)	100.557 (15)
*V* (Å^3^)	415.47 (13)	415.47 (13)	415.47 (13)
Radiation type	Mo *K*α	Mo *K*α	Mo *K*α
No. of reflections for cell measurement	514	514	514
θ range (°) for cell measurement	2.8–22.4	2.8–22.4	2.8–22.4
μ (mm^−1^)	19.29	19.29	19.29

Data collection [total experiment time = 32 hours, exposure time = 60 seconds, *F*_2_/σ(*F*_2_) = 10, data completeness = 58% (inf = 0.8 Å)]			
Absorption correction	Gaussian	Gaussian	Sphere
*T*_min_, *T*_max_	0.361, 0.434	0.361, 0.434	0.638, 0.645
No. of measured, independent and observed [*I* > 2σ(*I*)] reflections	2177, 550, 323	2116, 531, 313	2125, 531, 319
*R*_int_	0.105	0.103	0.102
(sin θ/λ)_max_ (Å^−1^)	0.663	0.663	0.663
Range of *h*, *k*, *l*	*h* = −5→5, *k* = −12→11, *l* = −10→10	*h* = −5→5, *k* = −12→11, *l* = −10→10	*h* = −5→5, *k* = −12→11, *l* = −10→10

Refinement			
*R*[*F* ^2^ > 2σ(*F* ^2^)], *wR*(*F* ^2^), *S*	0.097, 0.345, 1.17	0.073, 0.177, 1.04	0.071, 0.167, 1.03
No. of reflections	550	531	531
No. of parameters	31	31	31
(Δ/σ)_max_	< 0.001	< 0.001	< 0.001
Δρ_max_, Δρ_min_ (e Å^−3^)	2.65, −2.90	1.03, −0.88	0.95, −0.82

## supplementary crystallographic information

### 1,2,4,5-Tetrabromobenzene (Ag-Absorb7-raw_a) Crystal data

**Table:**

C_6_H_2_Br_4_	*F*(000) = 356
*M**_r_* = 393.72	*D*_x_ = 3.149 Mg m^−^^3^
Monoclinic, *P*2_1_/*n*	Ag *K*α radiation, λ = 0.56087 Å
*a* = 3.9390 (9) Å	Cell parameters from 748 reflections
*b* = 10.781 (4) Å	θ = 2.2–22.9°
*c* = 9.944 (4) Å	µ = 10.33 mm^−^^1^
β = 100.49 (3)°	*T* = 293 K
*V* = 415.2 (2) Å^3^	Block, colourless
*Z* = 2	0.18 × 0.07 × 0.01 mm

### 1,2,4,5-Tetrabromobenzene (Ag-Absorb7-raw_a) Data collection

**Table:**

XtaLAB Synergy, Dualflex, Pilatus 300K diffractometer	513 reflections with *I* > 2σ(*I*)
ω–scan	*R*_int_ = 0.048
Absorption correction: gaussian [CrysAlis PRO (Rigaku OD, 2016) and *ABSORB* (Angel *et al.*, 2007)]	θ_max_ = 26.7°, θ_min_ = 2.2°
*T*_min_ = 0.486, *T*_max_ = 0.562	*h* = −5→6
2503 measured reflections	*k* = −14→14
893 independent reflections	*l* = −11→12

### 1,2,4,5-Tetrabromobenzene (Ag-Absorb7-raw_a) Refinement

**Table:**

Refinement on *F*^2^	0 restraints
Least-squares matrix: full	Hydrogen site location: inferred from neighbouring sites
*R*[*F*^2^ > 2σ(*F*^2^)] = 0.047	H-atom parameters constrained
*wR*(*F*^2^) = 0.206	*w* = 1/[σ^2^(*F*_o_^2^) + (0.1247*P*)^2^] where *P* = (*F*_o_^2^ + 2*F*_c_^2^)/3
*S* = 1.02	(Δ/σ)_max_ = 0.014
893 reflections	Δρ_max_ = 1.55 e Å^−^^3^
46 parameters	Δρ_min_ = −1.48 e Å^−^^3^

### 1,2,4,5-Tetrabromobenzene (Ag-Absorb7-raw_a) Special details

**Table:**

Geometry. All esds (except the esd in the dihedral angle between two l.s. planes) are estimated using the full covariance matrix. The cell esds are taken into account individually in the estimation of esds in distances, angles and torsion angles; correlations between esds in cell parameters are only used when they are defined by crystal symmetry. An approximate (isotropic) treatment of cell esds is used for estimating esds involving l.s. planes.

### 1,2,4,5-Tetrabromobenzene (Ag-Absorb7-raw_a) Fractional atomic coordinates and isotropic or equivalent isotropic displacement parameters (Å^2^)

**Table:**

	*x*	*y*	*z*	*U*_iso_*/*U*_eq_	
Br1	0.5830 (3)	0.79104 (12)	0.59419 (15)	0.0407 (5)	
Br2	0.3468 (3)	0.57488 (12)	0.80180 (13)	0.0377 (5)	
C1	0.533 (3)	0.6214 (13)	0.5426 (15)	0.035 (3)	
C2	0.441 (3)	0.5314 (12)	0.6295 (13)	0.029 (3)	
C3	0.402 (3)	0.4136 (10)	0.5879 (15)	0.034 (3)	
H3	0.331794	0.354165	0.644830	0.041*	

### 1,2,4,5-Tetrabromobenzene (Ag-Absorb7-raw_a) Atomic displacement parameters (Å^2^)

**Table:**

	*U*^11^	*U*^22^	*U*^33^	*U*^12^	*U*^13^	*U*^23^
Br1	0.0582 (8)	0.0262 (10)	0.0386 (12)	−0.0032 (5)	0.0110 (6)	−0.0034 (4)
Br2	0.0498 (7)	0.0380 (11)	0.0280 (11)	0.0009 (5)	0.0142 (6)	−0.0026 (4)
C1	0.023 (5)	0.040 (10)	0.038 (10)	−0.008 (5)	−0.001 (5)	−0.009 (5)
C2	0.028 (5)	0.041 (10)	0.021 (10)	0.001 (5)	0.011 (5)	−0.008 (4)
C3	0.023 (5)	0.002 (8)	0.079 (12)	−0.008 (4)	0.014 (5)	0.001 (4)

### 1,2,4,5-Tetrabromobenzene (Ag-Absorb7-raw_a) Geometric parameters (Å, º)

**Table:**

Br1—C1	1.900 (14)	C1—C3^i^	1.419 (19)
Br2—C2	1.878 (12)	C2—C3	1.336 (16)
C1—C2	1.389 (19)	C3—H3	0.9300
			
C2—C1—C3^i^	119.5 (12)	C1—C2—Br2	120.7 (10)
C2—C1—Br1	122.0 (11)	C2—C3—C1^i^	120.4 (11)
C3^i^—C1—Br1	118.4 (10)	C2—C3—H3	119.8
C3—C2—C1	120.0 (13)	C1^i^—C3—H3	119.8
C3—C2—Br2	119.3 (10)		
			
C3^i^—C1—C2—C3	−2.6 (19)	Br1—C1—C2—Br2	2.2 (14)
Br1—C1—C2—C3	178.7 (8)	C1—C2—C3—C1^i^	2.7 (19)
C3^i^—C1—C2—Br2	−179.1 (9)	Br2—C2—C3—C1^i^	179.2 (9)

Symmetry code: (i) −*x*+1, −*y*+1, −*z*+1.

### 1,2,4,5-tetrabromobenzene (Ag-Absorb7_b) Crystal data

**Table:**

C_6_H_2_Br_4_	*F*(000) = 356
*M**_r_* = 393.72	*D*_x_ = 3.149 Mg m^−^^3^
Monoclinic, *P*2_1_/*n*	Ag *K*α radiation, λ = 0.56087 Å
*a* = 3.9390 (9) Å	Cell parameters from 748 reflections
*b* = 10.781 (4) Å	θ = 2.2–22.9°
*c* = 9.944 (4) Å	µ = 10.33 mm^−^^1^
β = 100.49 (3)°	*T* = 293 K
*V* = 415.2 (2) Å^3^	Block, colourless
*Z* = 2	0.18 × 0.07 × 0.01 mm

### 1,2,4,5-tetrabromobenzene (Ag-Absorb7_b) Data collection

**Table:**

XtaLAB Synergy, Dualflex, Pilatus 300K diffractometer	496 reflections with *I* > 2σ(*I*)
ω–scan	*R*_int_ = 0.048
Absorption correction: gaussian (CrysAlisPro; Rigaku OD, 2016) and (*ABSORB*; Angel *et al.*, 2007)	θ_max_ = 26.7°, θ_min_ = 2.2°
*T*_min_ = 0.486, *T*_max_ = 0.562	*h* = −5→6
2445 measured reflections	*k* = −14→14
870 independent reflections	*l* = −11→12

### 1,2,4,5-tetrabromobenzene (Ag-Absorb7_b) Refinement

**Table:**

Refinement on *F*^2^	0 restraints
Least-squares matrix: full	Hydrogen site location: inferred from neighbouring sites
*R*[*F*^2^ > 2σ(*F*^2^)] = 0.037	H-atom parameters constrained
*wR*(*F*^2^) = 0.073	*w* = 1/[σ^2^(*F*_o_^2^) + (0.023*P*)^2^] where *P* = (*F*_o_^2^ + 2*F*_c_^2^)/3
*S* = 0.93	(Δ/σ)_max_ = 0.001
870 reflections	Δρ_max_ = 0.54 e Å^−^^3^
46 parameters	Δρ_min_ = −0.54 e Å^−^^3^

### 1,2,4,5-tetrabromobenzene (Ag-Absorb7_b) Special details

**Table:**

Geometry. All esds (except the esd in the dihedral angle between two l.s. planes) are estimated using the full covariance matrix. The cell esds are taken into account individually in the estimation of esds in distances, angles and torsion angles; correlations between esds in cell parameters are only used when they are defined by crystal symmetry. An approximate (isotropic) treatment of cell esds is used for estimating esds involving l.s. planes.

### 1,2,4,5-tetrabromobenzene (Ag-Absorb7_b) Fractional atomic coordinates and isotropic or equivalent isotropic displacement parameters (Å^2^)

**Table:**

	*x*	*y*	*z*	*U*_iso_*/*U*_eq_	
Br1	0.58340 (14)	0.79093 (6)	0.59432 (7)	0.0409 (2)	
Br2	0.34699 (14)	0.57466 (6)	0.80194 (7)	0.0380 (2)	
C1	0.5332 (12)	0.6218 (6)	0.5414 (7)	0.0314 (15)	
C2	0.4386 (12)	0.5325 (5)	0.6290 (6)	0.0276 (14)	
C3	0.4002 (12)	0.4113 (5)	0.5857 (7)	0.0297 (14)	
H3	0.329328	0.351372	0.641933	0.036*	

### 1,2,4,5-tetrabromobenzene (Ag-Absorb7_b) Atomic displacement parameters (Å^2^)

**Table:**

	*U*^11^	*U*^22^	*U*^33^	*U*^12^	*U*^13^	*U*^23^
Br1	0.0583 (4)	0.0255 (5)	0.0399 (6)	−0.0036 (3)	0.0115 (3)	−0.0034 (2)
Br2	0.0502 (3)	0.0372 (5)	0.0291 (5)	0.0015 (3)	0.0139 (3)	−0.0027 (2)
C1	0.026 (2)	0.020 (5)	0.046 (6)	0.003 (2)	0.001 (3)	−0.004 (2)
C2	0.027 (3)	0.036 (5)	0.022 (6)	0.004 (3)	0.010 (3)	−0.002 (2)
C3	0.033 (3)	0.017 (5)	0.041 (6)	−0.004 (3)	0.013 (3)	0.005 (2)

### 1,2,4,5-tetrabromobenzene (Ag-Absorb7_b) Geometric parameters (Å, º)

**Table:**

Br1—C1	1.899 (6)	C1—C2	1.394 (7)
Br2—C2	1.876 (6)	C2—C3	1.377 (8)
C1—C3^i^	1.382 (8)	C3—H3	0.9300
			
C3^i^—C1—C2	120.7 (6)	C1—C2—Br2	121.6 (5)
C3^i^—C1—Br1	118.2 (4)	C2—C3—C1^i^	120.2 (5)
C2—C1—Br1	121.0 (5)	C2—C3—H3	119.9
C3—C2—C1	119.1 (6)	C1^i^—C3—H3	119.9
C3—C2—Br2	119.2 (4)		
			
C3^i^—C1—C2—C3	−2.3 (9)	Br1—C1—C2—Br2	1.3 (6)
Br1—C1—C2—C3	179.1 (4)	C1—C2—C3—C1^i^	2.2 (9)
C3^i^—C1—C2—Br2	180.0 (4)	Br2—C2—C3—C1^i^	−179.9 (4)

Symmetry code: (i) −*x*+1, −*y*+1, −*z*+1.

### 1,2,4,5-tetrabromobenzene (Ag-CAsphere_c) Crystal data

**Table:**

C_6_H_2_Br_4_	*F*(000) = 356
*M**_r_* = 393.72	*D*_x_ = 3.149 Mg m^−^^3^
Monoclinic, *P*2_1_/*n*	Ag *K*α radiation, λ = 0.56087 Å
*a* = 3.9390 (9) Å	Cell parameters from 748 reflections
*b* = 10.781 (4) Å	θ = 2.2–22.9°
*c* = 9.944 (4) Å	µ = 10.33 mm^−^^1^
β = 100.49 (3)°	*T* = 293 K
*V* = 415.2 (2) Å^3^	Block, colourless
*Z* = 2	0.18 × 0.07 × 0.01 mm

### 1,2,4,5-tetrabromobenzene (Ag-CAsphere_c) Data collection

**Table:**

XtaLAB Synergy, Dualflex, Pilatus 300K diffractometer	494 reflections with *I* > 2σ(*I*)
ω–scan	*R*_int_ = 0.050
Absorption correction: for a sphere (CrysAlisPro; Rigaku OD, 2016)	θ_max_ = 26.7°, θ_min_ = 2.2°
*T*_min_ = 0.638, *T*_max_ = 0.645	*h* = −5→6
2453 measured reflections	*k* = −14→14
870 independent reflections	*l* = −11→12

### 1,2,4,5-tetrabromobenzene (Ag-CAsphere_c) Refinement

**Table:**

Refinement on *F*^2^	0 restraints
Least-squares matrix: full	Hydrogen site location: inferred from neighbouring sites
*R*[*F*^2^ > 2σ(*F*^2^)] = 0.037	H-atom parameters constrained
*wR*(*F*^2^) = 0.071	*w* = 1/[σ^2^(*F*_o_^2^) + (0.023*P*)^2^] where *P* = (*F*_o_^2^ + 2*F*_c_^2^)/3
*S* = 0.91	(Δ/σ)_max_ < 0.001
870 reflections	Δρ_max_ = 0.53 e Å^−^^3^
46 parameters	Δρ_min_ = −0.49 e Å^−^^3^

### 1,2,4,5-tetrabromobenzene (Ag-CAsphere_c) Special details

**Table:**

Geometry. All esds (except the esd in the dihedral angle between two l.s. planes) are estimated using the full covariance matrix. The cell esds are taken into account individually in the estimation of esds in distances, angles and torsion angles; correlations between esds in cell parameters are only used when they are defined by crystal symmetry. An approximate (isotropic) treatment of cell esds is used for estimating esds involving l.s. planes.

### 1,2,4,5-tetrabromobenzene (Ag-CAsphere_c) Fractional atomic coordinates and isotropic or equivalent isotropic displacement parameters (Å^2^)

**Table:**

	*x*	*y*	*z*	*U*_iso_*/*U*_eq_	
Br1	0.58346 (14)	0.79099 (6)	0.59430 (7)	0.0412 (2)	
Br2	0.34702 (14)	0.57465 (6)	0.80195 (7)	0.0383 (2)	
C1	0.5329 (12)	0.6222 (5)	0.5413 (7)	0.0315 (14)	
C2	0.4384 (12)	0.5328 (5)	0.6291 (6)	0.0284 (14)	
C3	0.4001 (12)	0.4113 (5)	0.5855 (7)	0.0305 (14)	
H3	0.328780	0.351531	0.641771	0.037*	

### 1,2,4,5-tetrabromobenzene (Ag-CAsphere_c) Atomic displacement parameters (Å^2^)

**Table:**

	*U*^11^	*U*^22^	*U*^33^	*U*^12^	*U*^13^	*U*^23^
Br1	0.0587 (4)	0.0267 (5)	0.0392 (6)	−0.0037 (3)	0.0116 (3)	−0.0034 (2)
Br2	0.0506 (3)	0.0382 (5)	0.0286 (5)	0.0014 (3)	0.0140 (3)	−0.0026 (2)
C1	0.026 (2)	0.022 (5)	0.045 (6)	0.003 (2)	0.001 (3)	−0.004 (2)
C2	0.028 (3)	0.037 (5)	0.022 (6)	0.004 (3)	0.009 (3)	−0.002 (2)
C3	0.033 (3)	0.020 (5)	0.041 (6)	−0.004 (3)	0.014 (3)	0.005 (2)

### 1,2,4,5-tetrabromobenzene (Ag-CAsphere_c) Geometric parameters (Å, º)

**Table:**

Br1—C1	1.895 (6)	C1—C2	1.395 (7)
Br2—C2	1.875 (6)	C2—C3	1.379 (7)
C1—C3^i^	1.382 (8)	C3—H3	0.9300
			
C3^i^—C1—C2	120.5 (6)	C1—C2—Br2	121.7 (5)
C3^i^—C1—Br1	118.5 (4)	C2—C3—C1^i^	120.4 (5)
C2—C1—Br1	121.1 (5)	C2—C3—H3	119.8
C3—C2—C1	119.0 (6)	C1^i^—C3—H3	119.8
C3—C2—Br2	119.3 (4)		
			
C3^i^—C1—C2—C3	−2.4 (9)	Br1—C1—C2—Br2	1.4 (6)
Br1—C1—C2—C3	179.1 (4)	C1—C2—C3—C1^i^	2.4 (9)
C3^i^—C1—C2—Br2	179.8 (4)	Br2—C2—C3—C1^i^	−179.7 (4)

Symmetry code: (i) −*x*+1, −*y*+1, −*z*+1.

### 1,2,4,5-tetrabromobenzene (Mo-Absorb7-raw_d) Crystal data

**Table:**

C_6_H_2_Br_4_	*F*(000) = 356
*M**_r_* = 393.72	*D*_x_ = 3.147 Mg m^−^^3^
Monoclinic, *P*2_1_/*n*	Mo *K*α radiation, λ = 0.71073 Å
*a* = 3.9431 (5) Å	Cell parameters from 514 reflections
*b* = 10.7566 (18) Å	θ = 2.8–22.4°
*c* = 9.964 (2) Å	µ = 19.29 mm^−^^1^
β = 100.557 (15)°	*T* = 293 K
*V* = 415.47 (13) Å^3^	Block, colourless
*Z* = 2	0.18 × 0.07 × 0.01 mm

### 1,2,4,5-tetrabromobenzene (Mo-Absorb7-raw_d) Data collection

**Table:**

Xcalibur, Ruby, Gemini R Ultra diffractometer	323 reflections with *I* > 2σ(*I*)
ω–scan	*R*_int_ = 0.105
Absorption correction: gaussian (CrysAlisPro; Rigaku OD, 2016) and (*ABSORB*; Angel *et al.*, 2007)	θ_max_ = 28.1°, θ_min_ = 2.8°
*T*_min_ = 0.361, *T*_max_ = 0.434	*h* = −5→5
2177 measured reflections	*k* = −12→11
550 independent reflections	*l* = −10→10

### 1,2,4,5-tetrabromobenzene (Mo-Absorb7-raw_d) Refinement

**Table:**

Refinement on *F*^2^	0 restraints
Least-squares matrix: full	Hydrogen site location: inferred from neighbouring sites
*R*[*F*^2^ > 2σ(*F*^2^)] = 0.101	H-atom parameters constrained
*wR*(*F*^2^) = 0.347	*w* = 1/[σ^2^(*F*_o_^2^) + (0.2*P*)^2^] where *P* = (*F*_o_^2^ + 2*F*_c_^2^)/3
*S* = 1.19	(Δ/σ)_max_ = 0.089
550 reflections	Δρ_max_ = 2.65 e Å^−^^3^
46 parameters	Δρ_min_ = −2.89 e Å^−^^3^

### 1,2,4,5-tetrabromobenzene (Mo-Absorb7-raw_d) Special details

**Table:**

Geometry. All esds (except the esd in the dihedral angle between two l.s. planes) are estimated using the full covariance matrix. The cell esds are taken into account individually in the estimation of esds in distances, angles and torsion angles; correlations between esds in cell parameters are only used when they are defined by crystal symmetry. An approximate (isotropic) treatment of cell esds is used for estimating esds involving l.s. planes.

### 1,2,4,5-tetrabromobenzene (Mo-Absorb7-raw_d) Fractional atomic coordinates and isotropic or equivalent isotropic displacement parameters (Å^2^)

**Table:**

	*x*	*y*	*z*	*U*_iso_*/*U*_eq_	
Br1	0.5857 (8)	0.7915 (3)	0.5946 (4)	0.0384 (13)	
Br2	0.3478 (7)	0.5745 (3)	0.8014 (4)	0.0363 (13)	
C1	0.540 (6)	0.607 (5)	0.555 (6)	0.09 (2)	
C2	0.430 (7)	0.535 (3)	0.630 (4)	0.024 (8)	
C3	0.404 (7)	0.416 (3)	0.587 (4)	0.040 (10)	
H3	0.354750	0.351195	0.642577	0.048*	

### 1,2,4,5-tetrabromobenzene (Mo-Absorb7-raw_d) Atomic displacement parameters (Å^2^)

**Table:**

	*U*^11^	*U*^22^	*U*^33^	*U*^12^	*U*^13^	*U*^23^
Br1	0.053 (2)	0.017 (3)	0.046 (4)	−0.0029 (13)	0.0113 (19)	−0.0039 (12)
Br2	0.047 (2)	0.023 (3)	0.042 (4)	0.0010 (14)	0.0166 (19)	−0.0033 (12)
C1	0.004 (12)	0.08 (4)	0.18 (6)	−0.008 (17)	0.01 (2)	−0.12 (4)
C2	0.023 (14)	0.02 (3)	0.04 (3)	−0.002 (13)	0.019 (16)	0.002 (12)
C3	0.022 (14)	0.05 (3)	0.04 (4)	0.023 (15)	0.003 (16)	0.011 (16)

### 1,2,4,5-tetrabromobenzene (Mo-Absorb7-raw_d) Geometric parameters (Å, º)

**Table:**

Br1—C1	2.03 (4)	C1—C3^i^	1.49 (6)
Br2—C2	1.84 (3)	C2—C3	1.36 (4)
C1—C2	1.20 (7)	C3—H3	0.9300
			
C2—C1—C3^i^	128 (3)	C3—C2—Br2	120 (2)
C2—C1—Br1	122 (3)	C2—C3—C1^i^	116 (3)
C3^i^—C1—Br1	109 (4)	C2—C3—H3	122.0
C1—C2—C3	115 (4)	C1^i^—C3—H3	122.0
C1—C2—Br2	125 (3)		
			
C3^i^—C1—C2—C3	11 (6)	Br1—C1—C2—Br2	−10 (5)
Br1—C1—C2—C3	178 (2)	C1—C2—C3—C1^i^	−10 (5)
C3^i^—C1—C2—Br2	−176 (3)	Br2—C2—C3—C1^i^	177 (2)

Symmetry code: (i) −*x*+1, −*y*+1, −*z*+1.

### 1,2,4,5-tetrabromobenzene (Mo-Absorb7_e) Crystal data

**Table:**

C_6_H_2_Br_4_	*F*(000) = 356
*M**_r_* = 393.72	*D*_x_ = 3.147 Mg m^−^^3^
Monoclinic, *P*2_1_/*n*	Mo *K*α radiation, λ = 0.71073 Å
*a* = 3.9431 (5) Å	Cell parameters from 514 reflections
*b* = 10.7566 (18) Å	θ = 2.8–22.4°
*c* = 9.964 (2) Å	µ = 19.29 mm^−^^1^
β = 100.557 (15)°	*T* = 293 K
*V* = 415.47 (13) Å^3^	Block, colourless
*Z* = 2	0.18 × 0.07 × 0.01 mm

### 1,2,4,5-tetrabromobenzene (Mo-Absorb7_e) Data collection

**Table:**

Xcalibur, Ruby, Gemini R Ultra diffractometer	313 reflections with *I* > 2σ(*I*)
ω–scan	*R*_int_ = 0.103
Absorption correction: gaussian (CrysAlisPro; Rigaku OD, 2016) and (*ABSORB*; Angel *et al.*, 2007)	θ_max_ = 28.1°, θ_min_ = 2.8°
*T*_min_ = 0.361, *T*_max_ = 0.434	*h* = −5→5
2116 measured reflections	*k* = −12→11
531 independent reflections	*l* = −10→10

### 1,2,4,5-tetrabromobenzene (Mo-Absorb7_e) Refinement

**Table:**

Refinement on *F*^2^	0 restraints
Least-squares matrix: full	Hydrogen site location: inferred from neighbouring sites
*R*[*F*^2^ > 2σ(*F*^2^)] = 0.071	H-atom parameters constrained
*wR*(*F*^2^) = 0.169	*w* = 1/[σ^2^(*F*_o_^2^) + (0.0743*P*)^2^] where *P* = (*F*_o_^2^ + 2*F*_c_^2^)/3
*S* = 1.05	(Δ/σ)_max_ = 0.592
531 reflections	Δρ_max_ = 1.04 e Å^−^^3^
46 parameters	Δρ_min_ = −0.89 e Å^−^^3^

### 1,2,4,5-tetrabromobenzene (Mo-Absorb7_e) Special details

**Table:**

Geometry. All esds (except the esd in the dihedral angle between two l.s. planes) are estimated using the full covariance matrix. The cell esds are taken into account individually in the estimation of esds in distances, angles and torsion angles; correlations between esds in cell parameters are only used when they are defined by crystal symmetry. An approximate (isotropic) treatment of cell esds is used for estimating esds involving l.s. planes.

### 1,2,4,5-tetrabromobenzene (Mo-Absorb7_e) Fractional atomic coordinates and isotropic or equivalent isotropic displacement parameters (Å^2^)

**Table:**

	*x*	*y*	*z*	*U*_iso_*/*U*_eq_	
Br1	0.5856 (4)	0.79126 (19)	0.5942 (2)	0.0406 (8)	
Br2	0.3480 (4)	0.57453 (18)	0.8014 (2)	0.0377 (7)	
C1	0.537 (3)	0.6206 (17)	0.541 (2)	0.026 (5)	
C2	0.440 (4)	0.5357 (17)	0.632 (2)	0.030 (5)	
C3	0.405 (4)	0.4135 (14)	0.5861 (18)	0.020 (4)	
H3	0.341002	0.352930	0.643177	0.024*	

### 1,2,4,5-tetrabromobenzene (Mo-Absorb7_e) Atomic displacement parameters (Å^2^)

**Table:**

	*U*^11^	*U*^22^	*U*^33^	*U*^12^	*U*^13^	*U*^23^
Br1	0.0565 (13)	0.021 (2)	0.046 (2)	−0.0034 (9)	0.0131 (12)	−0.0039 (8)
Br2	0.0503 (12)	0.032 (2)	0.034 (2)	0.0011 (9)	0.0154 (11)	−0.0028 (7)
C1	0.022 (8)	0.010 (19)	0.04 (2)	−0.008 (7)	0.001 (9)	0.000 (7)
C2	0.031 (9)	0.000 (19)	0.07 (2)	0.006 (8)	0.023 (11)	0.003 (7)
C3	0.035 (9)	0.006 (15)	0.020 (18)	−0.003 (7)	0.009 (9)	0.011 (6)

### 1,2,4,5-tetrabromobenzene (Mo-Absorb7_e) Geometric parameters (Å, º)

**Table:**

Br1—C1	1.911 (19)	C1—C2	1.39 (2)
Br2—C2	1.84 (2)	C2—C3	1.39 (2)
C1—C3^i^	1.38 (3)	C3—H3	0.9300
			
C3^i^—C1—C2	122.4 (19)	C3—C2—Br2	119.3 (13)
C3^i^—C1—Br1	119.1 (12)	C1^i^—C3—C2	121.9 (14)
C2—C1—Br1	118.5 (17)	C1^i^—C3—H3	119.0
C1—C2—C3	116 (2)	C2—C3—H3	119.0
C1—C2—Br2	125.0 (17)		
			
C3^i^—C1—C2—C3	0 (3)	Br1—C1—C2—Br2	0.5 (18)
Br1—C1—C2—C3	179.2 (10)	C1—C2—C3—C1^i^	0 (2)
C3^i^—C1—C2—Br2	−178.5 (13)	Br2—C2—C3—C1^i^	178.6 (13)

Symmetry code: (i) −*x*+1, −*y*+1, −*z*+1.

### 1,2,4,5-tetrabromobenzene (Mo-CAsphere_f) Crystal data

**Table:**

C_6_H_2_Br_4_	*F*(000) = 356
*M**_r_* = 393.72	*D*_x_ = 3.147 Mg m^−^^3^
Monoclinic, *P*2_1_/*n*	Mo *K*α radiation, λ = 0.71073 Å
*a* = 3.9431 (5) Å	Cell parameters from 514 reflections
*b* = 10.7566 (18) Å	θ = 2.8–22.4°
*c* = 9.964 (2) Å	µ = 19.29 mm^−^^1^
β = 100.557 (15)°	*T* = 293 K
*V* = 415.47 (13) Å^3^	Block, colourless
*Z* = 2	0.18 × 0.07 × 0.01 × 0.03 (radius) mm

### 1,2,4,5-tetrabromobenzene (Mo-CAsphere_f) Data collection

**Table:**

Xcalibur, Ruby, Gemini R Ultra diffractometer	319 reflections with *I* > 2σ(*I*)
ω–scan	*R*_int_ = 0.102
Absorption correction: for a sphere (CrysAlisPro; Rigaku OD, 2016)	θ_max_ = 28.1°, θ_min_ = 2.8°
*T*_min_ = 0.638, *T*_max_ = 0.645	*h* = −5→5
2125 measured reflections	*k* = −12→11
531 independent reflections	*l* = −10→10

### 1,2,4,5-tetrabromobenzene (Mo-CAsphere_f) Refinement

**Table:**

Refinement on *F*^2^	0 restraints
Least-squares matrix: full	Hydrogen site location: inferred from neighbouring sites
*R*[*F*^2^ > 2σ(*F*^2^)] = 0.069	H-atom parameters constrained
*wR*(*F*^2^) = 0.157	*w* = 1/[σ^2^(*F*_o_^2^) + (0.0698*P*)^2^] where *P* = (*F*_o_^2^ + 2*F*_c_^2^)/3
*S* = 1.05	(Δ/σ)_max_ = 0.523
531 reflections	Δρ_max_ = 0.93 e Å^−^^3^
46 parameters	Δρ_min_ = −0.83 e Å^−^^3^

### 1,2,4,5-tetrabromobenzene (Mo-CAsphere_f) Special details

**Table:**

Geometry. All esds (except the esd in the dihedral angle between two l.s. planes) are estimated using the full covariance matrix. The cell esds are taken into account individually in the estimation of esds in distances, angles and torsion angles; correlations between esds in cell parameters are only used when they are defined by crystal symmetry. An approximate (isotropic) treatment of cell esds is used for estimating esds involving l.s. planes.

### 1,2,4,5-tetrabromobenzene (Mo-CAsphere_f) Fractional atomic coordinates and isotropic or equivalent isotropic displacement parameters (Å^2^)

**Table:**

	*x*	*y*	*z*	*U*_iso_*/*U*_eq_	
Br1	0.5853 (4)	0.79122 (17)	0.5942 (2)	0.0412 (7)	
Br2	0.3482 (4)	0.57458 (17)	0.8016 (2)	0.0387 (7)	
C1	0.537 (3)	0.6200 (15)	0.5426 (19)	0.024 (4)	
C2	0.436 (4)	0.5349 (16)	0.630 (2)	0.030 (5)	
C3	0.406 (3)	0.4125 (14)	0.5870 (17)	0.023 (4)	
H3	0.346632	0.351591	0.644834	0.027*	

### 1,2,4,5-tetrabromobenzene (Mo-CAsphere_f) Atomic displacement parameters (Å^2^)

**Table:**

	*U*^11^	*U*^22^	*U*^33^	*U*^12^	*U*^13^	*U*^23^
Br1	0.0580 (12)	0.0185 (19)	0.049 (2)	−0.0036 (8)	0.0148 (11)	−0.0039 (7)
Br2	0.0505 (11)	0.0301 (18)	0.039 (2)	0.0013 (8)	0.0170 (10)	−0.0032 (7)
C1	0.020 (7)	0.011 (17)	0.04 (2)	−0.006 (7)	−0.003 (8)	−0.007 (7)
C2	0.033 (8)	0.000 (17)	0.06 (2)	0.004 (7)	0.022 (10)	0.000 (7)
C3	0.031 (8)	0.010 (15)	0.029 (17)	0.000 (7)	0.008 (8)	0.017 (6)

### 1,2,4,5-tetrabromobenzene (Mo-CAsphere_f) Geometric parameters (Å, º)

**Table:**

Br1—C1	1.912 (17)	C1—C3^i^	1.40 (2)
Br2—C2	1.860 (18)	C2—C3	1.38 (2)
C1—C2	1.37 (2)	C3—H3	0.9300
			
C2—C1—C3^i^	122.2 (17)	C3—C2—Br2	118.7 (12)
C2—C1—Br1	120.1 (16)	C2—C3—C1^i^	120.2 (13)
C3^i^—C1—Br1	117.7 (12)	C2—C3—H3	119.9
C1—C2—C3	117.6 (18)	C1^i^—C3—H3	119.9
C1—C2—Br2	123.7 (15)		
			
C3^i^—C1—C2—C3	3 (2)	Br1—C1—C2—Br2	−1.2 (18)
Br1—C1—C2—C3	−179.9 (10)	C1—C2—C3—C1^i^	−2 (2)
C3^i^—C1—C2—Br2	−178.7 (11)	Br2—C2—C3—C1^i^	178.7 (11)

Symmetry code: (i) −*x*+1, −*y*+1, −*z*+1.

### 1,2,4,5-tetrabromobenzene (Mo-Absorb7-raw-Ciso_g) Crystal data

**Table:**

C_6_H_2_Br_4_	*F*(000) = 356
*M**_r_* = 393.72	*D*_x_ = 3.147 Mg m^−^^3^
Monoclinic, *P*2_1_/*n*	Mo *K*α radiation, λ = 0.71073 Å
*a* = 3.9431 (5) Å	Cell parameters from 514 reflections
*b* = 10.7566 (18) Å	θ = 2.8–22.4°
*c* = 9.964 (2) Å	µ = 19.29 mm^−^^1^
β = 100.557 (15)°	*T* = 293 K
*V* = 415.47 (13) Å^3^	Block, colourless
*Z* = 2	0.18 × 0.07 × 0.01 mm

### 1,2,4,5-tetrabromobenzene (Mo-Absorb7-raw-Ciso_g) Data collection

**Table:**

Xcalibur, Ruby, Gemini R Ultra diffractometer	323 reflections with *I* > 2σ(*I*)
ω–scan	*R*_int_ = 0.105
Absorption correction: gaussian (CrysAlisPro; Rigaku OD, 2016) and (*ABSORB*; Angel *et al.*, 2007)	θ_max_ = 28.1°, θ_min_ = 2.8°
*T*_min_ = 0.361, *T*_max_ = 0.434	*h* = −5→5
2177 measured reflections	*k* = −12→11
550 independent reflections	*l* = −10→10

### 1,2,4,5-tetrabromobenzene (Mo-Absorb7-raw-Ciso_g) Refinement

**Table:**

Refinement on *F*^2^	0 restraints
Least-squares matrix: full	Hydrogen site location: inferred from neighbouring sites
*R*[*F*^2^ > 2σ(*F*^2^)] = 0.097	H-atom parameters constrained
*wR*(*F*^2^) = 0.345	*w* = 1/[σ^2^(*F*_o_^2^) + (0.2*P*)^2^] where *P* = (*F*_o_^2^ + 2*F*_c_^2^)/3
*S* = 1.17	(Δ/σ)_max_ < 0.001
550 reflections	Δρ_max_ = 2.65 e Å^−^^3^
31 parameters	Δρ_min_ = −2.90 e Å^−^^3^

### 1,2,4,5-tetrabromobenzene (Mo-Absorb7-raw-Ciso_g) Special details

**Table:**

Geometry. All esds (except the esd in the dihedral angle between two l.s. planes) are estimated using the full covariance matrix. The cell esds are taken into account individually in the estimation of esds in distances, angles and torsion angles; correlations between esds in cell parameters are only used when they are defined by crystal symmetry. An approximate (isotropic) treatment of cell esds is used for estimating esds involving l.s. planes.

### 1,2,4,5-tetrabromobenzene (Mo-Absorb7-raw-Ciso_g) Fractional atomic coordinates and isotropic or equivalent isotropic displacement parameters (Å^2^)

**Table:**

	*x*	*y*	*z*	*U*_iso_*/*U*_eq_	
Br1	0.5856 (7)	0.7911 (3)	0.5945 (4)	0.0376 (13)	
Br2	0.3477 (7)	0.5745 (3)	0.8014 (4)	0.0368 (13)	
C1	0.539 (7)	0.617 (3)	0.545 (4)	0.034 (7)*	
C2	0.440 (6)	0.535 (3)	0.632 (3)	0.022 (6)*	
C3	0.404 (7)	0.412 (3)	0.587 (4)	0.034 (7)*	
H3	0.343843	0.350504	0.643580	0.041*	

### 1,2,4,5-tetrabromobenzene (Mo-Absorb7-raw-Ciso_g) Atomic displacement parameters (Å^2^)

**Table:**

	*U*^11^	*U*^22^	*U*^33^	*U*^12^	*U*^13^	*U*^23^
Br1	0.053 (2)	0.016 (3)	0.044 (3)	−0.0026 (13)	0.0115 (18)	−0.0036 (12)
Br2	0.046 (2)	0.025 (3)	0.042 (4)	0.0015 (13)	0.0158 (18)	−0.0030 (11)

### 1,2,4,5-tetrabromobenzene (Mo-Absorb7-raw-Ciso_g) Geometric parameters (Å, º)

**Table:**

Br1—C1	1.94 (3)	C1—C3^i^	1.41 (5)
Br2—C2	1.84 (3)	C2—C3	1.39 (4)
C1—C2	1.34 (4)	C3—H3	0.9300
			
C2—C1—C3^i^	125 (3)	C3—C2—Br2	119 (2)
C2—C1—Br1	120 (3)	C2—C3—C1^i^	119 (3)
C3^i^—C1—Br1	115 (2)	C2—C3—H3	120.5
C1—C2—C3	116 (3)	C1^i^—C3—H3	120.5
C1—C2—Br2	125 (3)		
			
C3^i^—C1—C2—C3	2 (5)	Br1—C1—C2—Br2	−1 (3)
Br1—C1—C2—C3	178.5 (18)	C1—C2—C3—C1^i^	−2 (5)
C3^i^—C1—C2—Br2	−177 (2)	Br2—C2—C3—C1^i^	177 (2)

Symmetry code: (i) −*x*+1, −*y*+1, −*z*+1.

### 1,2,4,5-tetrabromobenzene (Mo-Absorb7-Ciso_h) Crystal data

**Table:**

C_6_H_2_Br_4_	*F*(000) = 356
*M**_r_* = 393.72	*D*_x_ = 3.147 Mg m^−^^3^
Monoclinic, *P*2_1_/*n*	Mo *K*α radiation, λ = 0.71073 Å
*a* = 3.9431 (5) Å	Cell parameters from 514 reflections
*b* = 10.7566 (18) Å	θ = 2.8–22.4°
*c* = 9.964 (2) Å	µ = 19.29 mm^−^^1^
β = 100.557 (15)°	*T* = 293 K
*V* = 415.47 (13) Å^3^	Block, colourless
*Z* = 2	0.18 × 0.07 × 0.01 mm

### 1,2,4,5-tetrabromobenzene (Mo-Absorb7-Ciso_h) Data collection

**Table:**

Xcalibur, Ruby, Gemini R Ultra diffractometer	313 reflections with *I* > 2σ(*I*)
ω–scan	*R*_int_ = 0.103
Absorption correction: gaussian (CrysAlisPro; Rigaku OD, 2016) and (*ABSORB*; Angel *et al.*, 2007)	θ_max_ = 28.1°, θ_min_ = 2.8°
*T*_min_ = 0.361, *T*_max_ = 0.434	*h* = −5→5
2116 measured reflections	*k* = −12→11
531 independent reflections	*l* = −10→10

### 1,2,4,5-tetrabromobenzene (Mo-Absorb7-Ciso_h) Refinement

**Table:**

Refinement on *F*^2^	0 restraints
Least-squares matrix: full	Hydrogen site location: inferred from neighbouring sites
*R*[*F*^2^ > 2σ(*F*^2^)] = 0.073	H-atom parameters constrained
*wR*(*F*^2^) = 0.177	*w* = 1/[σ^2^(*F*_o_^2^) + (0.0807*P*)^2^] where *P* = (*F*_o_^2^ + 2*F*_c_^2^)/3
*S* = 1.04	(Δ/σ)_max_ < 0.001
531 reflections	Δρ_max_ = 1.03 e Å^−^^3^
31 parameters	Δρ_min_ = −0.88 e Å^−^^3^

### 1,2,4,5-tetrabromobenzene (Mo-Absorb7-Ciso_h) Special details

**Table:**

Geometry. All esds (except the esd in the dihedral angle between two l.s. planes) are estimated using the full covariance matrix. The cell esds are taken into account individually in the estimation of esds in distances, angles and torsion angles; correlations between esds in cell parameters are only used when they are defined by crystal symmetry. An approximate (isotropic) treatment of cell esds is used for estimating esds involving l.s. planes.

### 1,2,4,5-tetrabromobenzene (Mo-Absorb7-Ciso_h) Fractional atomic coordinates and isotropic or equivalent isotropic displacement parameters (Å^2^)

**Table:**

	*x*	*y*	*z*	*U*_iso_*/*U*_eq_	
Br1	0.5856 (4)	0.79134 (18)	0.5943 (2)	0.0404 (8)	
Br2	0.3480 (4)	0.57458 (18)	0.8015 (2)	0.0376 (8)	
C1	0.536 (3)	0.6214 (16)	0.5414 (19)	0.026 (4)*	
C2	0.440 (4)	0.5358 (17)	0.6328 (19)	0.026 (4)*	
C3	0.405 (3)	0.4134 (15)	0.5857 (19)	0.022 (4)*	
H3	0.338150	0.352764	0.642273	0.026*	

### 1,2,4,5-tetrabromobenzene (Mo-Absorb7-Ciso_h) Atomic displacement parameters (Å^2^)

**Table:**

	*U*^11^	*U*^22^	*U*^33^	*U*^12^	*U*^13^	*U*^23^
Br1	0.0565 (13)	0.021 (2)	0.045 (2)	−0.0031 (9)	0.0129 (12)	−0.0038 (8)
Br2	0.0502 (12)	0.031 (2)	0.034 (2)	0.0014 (9)	0.0154 (11)	−0.0029 (7)

### 1,2,4,5-tetrabromobenzene (Mo-Absorb7-Ciso_h) Geometric parameters (Å, º)

**Table:**

Br1—C1	1.902 (18)	C1—C2	1.40 (2)
Br2—C2	1.831 (18)	C2—C3	1.40 (2)
C1—C3^i^	1.38 (2)	C3—H3	0.9300
			
C3^i^—C1—C2	122.1 (18)	C1—C2—Br2	124.9 (15)
C3^i^—C1—Br1	119.2 (12)	C1^i^—C3—C2	122.7 (15)
C2—C1—Br1	118.7 (15)	C1^i^—C3—H3	118.6
C3—C2—C1	115.2 (18)	C2—C3—H3	118.6
C3—C2—Br2	119.9 (12)		
			
C3^i^—C1—C2—C3	−1 (2)	Br1—C1—C2—Br2	1.3 (18)
Br1—C1—C2—C3	179.1 (10)	C1—C2—C3—C1^i^	1 (2)
C3^i^—C1—C2—Br2	−178.6 (12)	Br2—C2—C3—C1^i^	178.8 (12)

Symmetry code: (i) −*x*+1, −*y*+1, −*z*+1.

### 1,2,4,5-tetrabromobenzene (Mo-CAsphere-Ciso_i) Crystal data

**Table:**

C_6_H_2_Br_4_	*F*(000) = 356
*M**_r_* = 393.72	*D*_x_ = 3.147 Mg m^−^^3^
Monoclinic, *P*2_1_/*n*	Mo *K*α radiation, λ = 0.71073 Å
*a* = 3.9431 (5) Å	Cell parameters from 514 reflections
*b* = 10.7566 (18) Å	θ = 2.8–22.4°
*c* = 9.964 (2) Å	µ = 19.29 mm^−^^1^
β = 100.557 (15)°	*T* = 293 K
*V* = 415.47 (13) Å^3^	Block, colourless
*Z* = 2	0.18 × 0.07 × 0.01 × 0.03 (radius) mm

### 1,2,4,5-tetrabromobenzene (Mo-CAsphere-Ciso_i) Data collection

**Table:**

Xcalibur, Ruby, Gemini R Ultra diffractometer	319 reflections with *I* > 2σ(*I*)
ω–scan	*R*_int_ = 0.102
Absorption correction: for a sphere (CrysAlisPro; Rigaku OD, 2016)	θ_max_ = 28.1°, θ_min_ = 2.8°
*T*_min_ = 0.638, *T*_max_ = 0.645	*h* = −5→5
2125 measured reflections	*k* = −12→11
531 independent reflections	*l* = −10→10

### 1,2,4,5-tetrabromobenzene (Mo-CAsphere-Ciso_i) Refinement

**Table:**

Refinement on *F*^2^	0 restraints
Least-squares matrix: full	Hydrogen site location: inferred from neighbouring sites
*R*[*F*^2^ > 2σ(*F*^2^)] = 0.071	H-atom parameters constrained
*wR*(*F*^2^) = 0.167	*w* = 1/[σ^2^(*F*_o_^2^) + (0.078*P*)^2^] where *P* = (*F*_o_^2^ + 2*F*_c_^2^)/3
*S* = 1.03	(Δ/σ)_max_ < 0.001
531 reflections	Δρ_max_ = 0.95 e Å^−^^3^
31 parameters	Δρ_min_ = −0.82 e Å^−^^3^

### 1,2,4,5-tetrabromobenzene (Mo-CAsphere-Ciso_i) Special details

**Table:**

Geometry. All esds (except the esd in the dihedral angle between two l.s. planes) are estimated using the full covariance matrix. The cell esds are taken into account individually in the estimation of esds in distances, angles and torsion angles; correlations between esds in cell parameters are only used when they are defined by crystal symmetry. An approximate (isotropic) treatment of cell esds is used for estimating esds involving l.s. planes.

### 1,2,4,5-tetrabromobenzene (Mo-CAsphere-Ciso_i) Fractional atomic coordinates and isotropic or equivalent isotropic displacement parameters (Å^2^)

**Table:**

	*x*	*y*	*z*	*U*_iso_*/*U*_eq_	
Br1	0.5854 (4)	0.79127 (17)	0.5942 (2)	0.0411 (7)	
Br2	0.3482 (4)	0.57464 (17)	0.8016 (2)	0.0388 (7)	
C1	0.536 (3)	0.6208 (15)	0.5421 (18)	0.024 (4)*	
C2	0.438 (3)	0.5350 (15)	0.6310 (17)	0.026 (4)*	
C3	0.405 (3)	0.4119 (14)	0.5867 (18)	0.024 (4)*	
H3	0.342370	0.351016	0.643810	0.029*	

### 1,2,4,5-tetrabromobenzene (Mo-CAsphere-Ciso_i) Atomic displacement parameters (Å^2^)

**Table:**

	*U*^11^	*U*^22^	*U*^33^	*U*^12^	*U*^13^	*U*^23^
Br1	0.0581 (12)	0.0182 (19)	0.049 (2)	−0.0033 (8)	0.0145 (11)	−0.0038 (7)
Br2	0.0505 (11)	0.0300 (18)	0.039 (2)	0.0015 (8)	0.0168 (10)	−0.0033 (7)

### 1,2,4,5-tetrabromobenzene (Mo-CAsphere-Ciso_i) Geometric parameters (Å, º)

**Table:**

Br1—C1	1.906 (17)	C1—C3^i^	1.39 (2)
Br2—C2	1.848 (17)	C2—C3	1.39 (2)
C1—C2	1.38 (2)	C3—H3	0.9300
			
C2—C1—C3^i^	122.4 (17)	C3—C2—Br2	119.2 (11)
C2—C1—Br1	119.6 (14)	C1^i^—C3—C2	120.7 (14)
C3^i^—C1—Br1	117.9 (11)	C1^i^—C3—H3	119.6
C1—C2—C3	116.9 (16)	C2—C3—H3	119.6
C1—C2—Br2	123.9 (14)		
			
C3^i^—C1—C2—C3	1 (2)	Br1—C1—C2—Br2	0.0 (17)
Br1—C1—C2—C3	179.7 (9)	C1—C2—C3—C1^i^	−1 (2)
C3^i^—C1—C2—Br2	−178.8 (11)	Br2—C2—C3—C1^i^	178.8 (11)

Symmetry code: (i) −*x*+1, −*y*+1, −*z*+1.
